# Analysis of in-hospital mortality among people with and without diabetes in South Western Sydney public hospitals (2014–2017)

**DOI:** 10.1186/s12889-021-12120-w

**Published:** 2021-11-03

**Authors:** Tina Gao, Kingsley E. Agho, Milan K. Piya, David Simmons, Uchechukwu L. Osuagwu

**Affiliations:** 1 Translational Health Research Institute (THRI), School of Medicine, Western Sydney University, Campbelltown, NSW 2560 Australia; 2 School of Health Sciences, Western Sydney University, Campbelltown, NSW 2560 Australia; 3 African Vision Research Institute (AVRI), University of KwaZulu-Natal, Durban, 4041 South Africa; 4 Macarthur Diabetes Endocrinology and Metabolism Service, Camden and Campbelltown Hospital, Campbelltown, NSW 2560 Australia

**Keywords:** Mortality, Diabetes, Hospitalisations, Standard mortality ratio, Australia

## Abstract

**Background:**

Diabetes is a major public health problem affecting about 1.4 million Australians, especially in South Western Sydney, a hotspot of diabetes with higher than average rates for hospitalisations. The current understanding of the international burden of diabetes and related complications is poor and data on hospital outcomes and/or what common factors influence mortality rate in people with and without diabetes in Australia using a representative sample is lacking. This study determined in-hospital mortality rate and the factors associated among people with and without diabetes.

**Methods:**

Retrospective data for 554,421 adult inpatients was extracted from the population-based New South Wales (NSW) Admitted Patient Data over 3 financial years (from 1 July 2014–30 June 2015 to 1 July 2016–30 June 2017). The in-hospital mortality per 1000 admitted persons, standardised mortality ratios (SMR) were calculated. Binary logistic regression was performed, adjusting for potential covariates and co-morbidities for people with and without diabetes over three years.

**Results:**

Over three years, 8.7% (48,038 people) of admissions involved people with diabetes. This increased from 8.4% in 2014–15 to 8.9% in 2016–17 (*p* = 0.007). Across all age groups, in-hospital mortality rate was significantly greater in people with diabetes (20.6, 95% Confidence intervals CI 19.3–21.9 per 1000 persons) than those without diabetes (11.8, 95%CI 11.5–12.1) and more in men than women (23.1, 95%CI 21.2–25.0 vs 17.9, 95%CI 16.2–19.8) with diabetes. The SMR for those with and without diabetes were 3.13 (95%CI 1.78–4.48) and 1.79 (95%CI 0.77–2.82), respectively. There were similarities in the factors associated with in hospital mortality in both groups including: older age (> 54 years), male sex, marital status (divorced/widowed), length of stay in hospital (staying longer than 4 days), receiving intensive care in admission and being admitted due to primary respiratory and cardiovascular diagnoses. The odds of death in admission was increased in polymorbid patients without diabetes (28.68, 95%CI 23.49–35.02) but not in those with diabetes.

**Conclusions:**

In-patients with diabetes continue to have higher mortality rates than those without diabetes and the Australian population. Overall, similar factors influenced mortality rate in people with and without diabetes, but significantly more people with diabetes had two or more co-morbidities, suggesting that hospital mortality may be driven by those with pre-existing health/comorbidities. Urgent measures in primary care to prevent admissions among people with multiple co-morbidities are needed.

**Supplementary Information:**

The online version contains supplementary material available at 10.1186/s12889-021-12120-w.

## Introduction

Diabetes is a major public health problem affecting about 1.4 million Australians (4.9%) with an additional 300,000 with undiagnosed diabetes [1]. Diabetes was associated with about 1.2 million hospitalizations, representing 11% of all hospitalisations in Australia [1] and contributed to about 16,700 deaths in 2017–18 (10.5% of all deaths) [1]. Past studies showed higher mortality rate in people with diabetes than the general population of similar age [2], but more robust evidence on a representative sample are needed regarding the hospital outcomes of people living with diabetes [3]. People with type 1 diabetes were 2.4 times more likely to die, and type 2 diabetes were 1.4 times more likely to die than the general population [2]. Analysing the trends in mortality among Australians with diabetes between 1997 and 2010, a study found a significant decrease in mortality rates among males and females with type 1 and type 2 diabetes compared with the general population [4]. People with diabetes are also more likely to be admitted to hospital, stay in for longer [5] and be readmitted as an emergency [6] which increases the financial burden of diabetes that was estimated to be $14.6 billion in direct and indirect costs to the Australian economy [1].

South Western Sydney (SWS) is one of the most culturally diverse and rapidly growing populations in New South Wales (NSW) and covers both rural and suburban communities^.^ The region was chosen for this study because it has one of the highest prevalence of diabetes (6.7%) [1, 7] in Australia, poorer health outcomes among the residents, higher death rates and diabetes related hospitalisations [8] than other regions. Such high hospitalisation rates and mortality related to diabetes are likely to contribute to an increasingly burdened health care system unless measures to reduce diabetes morbidity and mortality are put into place.

Although some factors associated with increased mortality among inpatients with diabetes are described in the literature [9], including complications of diabetes [10] and specific patient characteristics [11], no study has analysed the common factors associated with inpatient mortality in people with and without diabetes in this region. Diabetes related complications of the vascular system, infections, chronic kidney disease [12], cardiovascular disease and cerebrovascular disease [13, 14], have been identified as leading causes of mortality in patients with diabetes but little is known about the role of such comorbidities (co-occurrence of one or more disorders/conditions) among inpatients without diabetes. Additionally, specific patient characteristics such as increasing age [15], gender [11], and socioeconomic disadvantage [16] were shown to be significant predictors of mortality. Identifying and comparing factors associated with mortality among inpatients in Australia, is required to provide an indication of the burden of disease and identify target groups for health promotion and diabetes management. As part of a project to improve diabetes care in this region and the need for in hospital data from a defined cohort for effective monitoring [4], this study was designed to determine the mortality rates of people with and without diabetes, admitted to public hospitals in SWS local health district (LHD) over a three-year period (2014–2017). In addition, the study examined the factors associated with mortality rates among inpatients with and without diabetes.

## Methods

### Data sources

The NSW Admitted Patient Data Collection (APDC) dataset [17] contains demographic and mortality related data for all inpatient admissions provided by state hospitals using International Classification of Diseases 10th revision, Australian Modification (ICD-10-AM). People are coded by their place of residence rather than admission hospital [17]. For this study, only public hospital APDC data of episodes of care were used [18]. The APDC dataset has been previously validated in people with and without diabetes [19–21]. For instance, when APDC was used as a standard for validating self-reported diabetes status of people in the 45 and up study, the authors were able to identify 48.3% more people with diabetes whose status were not previously reported in their dataset [22].

#### Ethical consideration

Approval for this study was obtained from the South Western Sydney Local Health District Human Ethics Research Committee as a quality improvement project (QA18/021).

### Study population

An extract of every hospital admission to a SWSLHD public hospital between 1 July 2014 and 30 June 2017 where the patient was 18 years or older on admission, and was in hospital for least 24 h, was taken from the APDC database. Data for this district were from six acute public hospitals including: Bankstown-Lidcombe Hospital, Bowral and District Hospital, Campbelltown and Camden Hospitals, Fairfield Hospital and Liverpool Hospital. Although inclusion of private hospitals would most accurately reflect shifts in volume between public and private hospitals, only data for all public hospital admissions within the period of study were used in this study because most acute admissions are in public hospitals. Data for inpatients in the psychiatric ward and rehabilitation ward admissions, were excluded. Between 2014 and 2017, NSW hospital admissions increased from 10,150,367 to 11,013,815 with approximately 60% of admissions occurring in public hospitals compared to 40% in private hospitals [23]. In the data registry, admissions were identified as relating to patients with diabetes if a code for diabetes (ICD -10 E10–E14) was included as a primary or secondary diagnosis. Data were available for residents from 6 out of 7 local government areas (LGAs) in SWS.

### Outcome variables

The dependent variable was death during admission in people with and without diabetes, which takes a binary form, such that death will be regarded as 1 if death occurs during admission or 0, if alive following admission.

### Covariates

The independent variables included: diabetes status, year of admission (three levels: July 2014 – June 2015, July 2015 – June 2016 and July 2016 – June 2017), demography which included age groups, sex, marital status, country of birth, place of residence (categorised as urban and peri-urban local government areas LGAs, based on the SWS Peri urban Network of Council action plan [24]), hospital health insurance cover (full or basic/no hospital cover), hospital admission and primary admission diagnosis determined based on the International Statistical Classification of Diseases and Related Health Problems. To understand the effect of polymorbidity on the mortality rates, we categorized the number of comorbidities into primary admission diagnosis plus either one comorbidity, or two/more comorbidities. Hospital admission included the length of stay (categorised as </= 4 days and > 4 days) [25, 26] in hospital and whether or not there was an ICU admission.

All hospital admissions < 24 h and those involving patients aged < 18 years were excluded as well as admissions relating to obstetrics as they have the potential to obscure findings for women of child-bearing age [11]. Hospital insurance status indicates whether the person receiving the inpatient service has a private health insurance cover or not at the time of admission. In *Australia*, *private health insurance* allows the person to be treated in public or private hospitals as a private patient with the doctor of his/her choice, help pay *for* health care costs that is not covered in the government Medicare system, such as physiotherapy, dental and optical, as well as access to some hospital services more quickly [23].

### Statistical analysis

The characteristics of patients with and without diabetes for each year of admission were presented using frequency tabulations. Mortality rate per 1000 admitted persons and their 95% Confidence Interval (CI) was calculated in the diabetes and the non-diabetes group for all independent variables using a direct method. It was calculated for each subcategory of participants as number of deaths divided by the number of admissions in that subcategory (sum of deaths and alive) multiplied by 1000 [27]. The standardized mortality ratio (SMR) was calculated using the Australian Bureau of Statistics (ABS) data [28] as the observed number of deaths divided by the expected number of deaths among adult Australians within the same period. An SMR < 1.0 indicates there were fewer than expected deaths, SMR = 1.0 indicates the number of observed deaths equals the number of expected deaths while SMR > 1.0 indicates there were more than expected deaths, in the study population (excess deaths). This gave a measure of relative mortality compared with all admissions with diabetes included in the analysis. This was followed by binary logistic regression which adjusted for potential covariates and co-morbidities for people with and without diabetes over three years. These were used to examine the factors associated with mortality among inpatients with and without diabetes and across the study population. The results were presented as odds ratios (OR) with their 95% CI. Analyses were performed using STATA version 14.1 (Stata Corporation 2015, College Station, TX, USA).

## Results

### Characteristics of people with and without diabetes in this study

Across the study period, there were 554,421 adult participants admitted to public hospitals in SWS between July 01, 2014 and June 30, 2017. Table 2 presents the characteristics of the study group for the three admission years. Over three years, 8.7% (48,038 people) of admissions involved those with diabetes, which increased significantly between 2014 and 15 and 2016–17 (8.4% versus 8.9%, *p* = 0.007). Among adults with diabetes, many were aged 65–74 years (26.5%), mostly males (52.3%), who were married including de-facto relationships (59.3%), born in Australia (37.9%) and lived in urban areas (65.3%). For those without diabetes, they were mostly females (52.8%), aged 18–54 years old (42.0%), married (56.8%), lived in urban areas (62.8%) and born in Australia (45.7%). Compared to the national Australian rate of 44%, only 9.8% of patients with diabetes and 7.5% of patients without diabetes had private health insurance [23].

About 2.5 and 1.0% of those with and without diabetes, respectively received intensive care in admission and majority of the participants stayed in hospital for less than 4 days (72.4% in the diabetes group and 84.8% in the non-diabetes group) over the study period. About 90% of admitted adults with diabetes and half of those without diabetes were polymorbid patients (i.e. had two or more comorbidities) and majority had diseases of the circulatory and digestive system, particularly in the diabetes group (Table 1).

**Table 1:** Demographic characteristics of adults with and without diabetes admitted to South Western Sydney Local Health District hospitals by admission year and diabetes status (2014–15, 2015–16 and 2016–17)

Characteristics	Diabetes			Non-diabetes		
Admission year	July 2014–15	July 2015–16	July 2016–17	July 2014–15	July 2015–16	July 2016–17
*N (%)*	14,661 (8.4)	16,219 (8.7)	17,158 (8.9)	160,265 (91.6)	169,943 (91.3)	176,175 (91.1)
Demography						
Age groups						
18–54	2854 (19.5)	3158 (19.5)	3294 (19.2)	67,148 (41.9)	71,341 (42.0)	75,438 (42.8)
55–64	2958 (20.2)	3248 (20.0)	3475 (20.3)	25,355 (15.8)	26,815 (15.8)	28,092 (16.0)
65–74	3894 (26.6)	4268 (26.3)	4572 (26.7)	29,496 (18.4)	31,508 (18.5)	31,865 (18.1)
75–84	3497 (23.9)	3811 (23.5)	3975 (23.2)	25,605 (16.0)	26,451 (15.6)	26,504 (15.0)
85+	1458 (9.9)	1734 (10.7)	1842 (10.7)	12,661 (7.9)	13,828 (8.1)	14,276 (8.1)
Sex						
Women	6916 (47.2)	7760 (47.9)	8250 (48.1)	85,502 (53.4)	89,714 (52.8)	91,902 (52.2)
Men	7745 (52.8)	8459 (52.2)	8908 (51.9)	74,763 (46.7)	80,225 (47.2)	84,271 (47.8)
Marital status						
Married/defacto	8644 (59.1)	9607 (59.4)	10,170 (59.4)	90,373 (56.6)	96,762 (57.0)	99,696 (56.7)
Widowed	4497 (30.7)	4961 (30.7)	5229 (30.5)	40,611 (25.4)	41,845 (24.7)	42,369 (24.1)
Single	1486 (10.2)	1616 (10.0)	1727 (10.1)	28,812 (18.0)	31,058 (18.3)	33,810 (19.2)
Place of birth						
Australia	5563 (38.0)	6073 (37.5)	6559 (38.3)	72,754 (45.4)	77,636 (45.7)	81,034 (46.1)
America	412 (2.8)	469 (2.9)	464 (2.7)	3931 (2.5)	4450 (2.6)	4365 (2.5)
Asia	3751 (25.7)	4416 (27.3)	4674 (27.3)	39,051 (24.4)	40,798 (24.0)	42,921 (24.4)
Africa	448 (3.1)	426 (2.6)	484 (2.8)	4610 (2.9)	5003 (2.9)	4920 (2.8)
Europe	3523 (24.1)	3673 (22.7)	3785 (22.1)	26,005 (16.2)	26,446 (15.6)	26,633 (15.1)
Pacific	927 (6.3)	1128 (7.0)	1163 (6.8)	13,743 (8.6)	15,389 (9.1)	16,067 (9.1)
Residence†						
Peri-urban	5076 (34.6)	5670 (35.0)	5913 (34.5)	59,239 (37.0)	62,658 (36.9)	66,209 (37.6)
Urban	9585 (65.4)	10,549 (65.0)	11,245 (65.5)	101,026 (63.0)	107,285 (63.1)	109,966 (62.4)
Hospital health insurance cover						
Full hospital cover	1410 (9.6)	1643 (10.1)	1691 (9.9)	11,262 (7.0)	12,924 (7.6)	13,061 (7.4)
No hospital cover	13,251 (90.4)	14,576 (89.9)	15,467 (90.1)	149,001 (93.0)	157,019 (92.4)	163,114 (92.6)
Episode length of stay (LOS)						
≤ 4 days	10,340 (70.5)	11,739 (72.4)	12,728 (74.2)	134,631 (84.0)	143,902 (84.7)	150,787 (85.6)
> 4 days	4321 (29.5)	4480 (27.6)	4430 (25.8)	25,634 (16.0)	26,041 (15.3)	25,388 (14.4)
LOS in days; median (IQR)	2 (1–5)	1 (1–5)	1 (1–5)	1 (1–3)	1 (1–3)	1 (1–2)
ICU admission						
No	14,300 (97.5)	15,824 (97.6)	16,692 (97.3)	158,591 (99.0)	168,235 (99.0)	174,178 (98.9)
Yes	360 (2.5)	393 (2.4)	463 (2.7)	1665 (1.0)	1697 (1.0)	1979 (1.1)
LOS in hours; median (IQR)	46 (21–90.5)	42 (19–78)	40 (17–77)	42 (16–94)	39 (15–88)	39 (16–80)
Primary admission diagnosis (ICD classification)						
Nervous system disease	359 (2.4)	421 (2.6)	425 (2.5)	2985 (1.9)	3436 (2.0)	3675 (2.1)
Respiratory system	1117 (7.6)	1177 (7.3)	1318 (7.7)	6881 (4.3)	7394 (4.4)	7888 (4.5)
Circulatory system	1889 (12.9)	2050 (12.6)	2115 (12.3)	8709 (5.4)	9223 (5.4)	9703 (5.5)
Musculoskeletal + connective system	673 (4.6)	837 (5.2)	866 (5.0)	4698 (2.9)	5292 (3.1)	5584 (3.2)
Skin + subcutaneous system	369 (2.5)	407 (2.5)	459 (2.7)	2537 (1.6)	3131 (1.8)	3158 (1.8)
Endocrine, nutritional + metabolic system	600 (4.1)	634 (3.9)	720 (4.2)	1896 (1.2)	1877 (1.1)	1877 (1.1)
Number of comorbidities						
Only primary admission diagnosis	40 (0.3)	25 (0.1)	27 (0.2)	63,138 (42.5)	64,021 (37.7)	65,467 (37.2)
Primary+ one comorbidity	2216 (15.1)	626 (3.9)	657 (3.8)	22,297 (13.9)	19,243 (11.3)	20,725 (11.8)
Primary+ 2/more comorbidities	12,404 (84.6)	16,218 (96.0)	16,473 (96.0)	69,786 (43.6)	86,648 (51.0)	89,937 (51.0)

*Data were for those who spent at least 1 day. (n, %) were used unless specified. ICD = International Statistical Classification of Diseases and Related Health Problems; ICU = intensive care unit; IQR = Interquartile range. †Peri urban includes Wollondilly, Camden, Wingecarribee Local Government Areas (LGA) while Urban includes Campbelltown, Fairfield, Bankstown-Lidcombe LGAs*

### Mortality rates in adults with and without diabetes on admission (2014–15 to 2016–17)

A total of 6981 adults died in hospital during the study period and 14.2% (*n* = 990) of all deaths involved people with diabetes. Table 2 presents the mortality rate per 1000 admitted persons and their 95% CIs in both groups over three years. The mortality rate across SWSLHD public hospitals was 12.6 (12.4–12.8) per 1000 admitted persons which was significantly higher in the diabetes 20.6 (95% CI 19.7–21.5) than non-diabetes 11.8 (95% CI 11.6–12.0) group. The mortality rate showed significant increase with age, reaching 48.3 (95% CI 43.9–52.6) and 48.1 (95% CI 46.5–49.6) per 1000 admitted persons aged 85 years and over, living with and without diabetes, respectively. Males had higher mortality rate than women but this was predominant in people with diabetes (23.1 [95% 21.7–24.4] versus 13.4 [95% 16.7–19.2], per 1000 admitted persons). In both groups, there were higher mortality rates among the previously married or divorced people, those who were born in European countries and had two or more comorbidities (Table 2).

**Table 2:** Mortality rate [per 1000 admitted persons (95% CI)] in adults with and without diabetes across South Western Sydney Local Health District (SWSLHD) public hospitals (2014–15, 2015–16 and 2016–17)

Variables	Diabetes	Non-Diabetes
Diabetes Status	20.6 (19.3–21.9)	11.8 (11.5–12.1)
Financial Year		
July 2014–15	22.2 (20.5–24.0)	12.4 (12.0–12.8)
July 2015–16	19.2 (17.8–20.8)	11.6 (11.2–11.9)
July 2016–17	20.5 (18.9–22.0)	11.6 (11.2–11.9)
Demography		
Age Groups		
18–54	6.2 (5.1–7.4)	2.5 (2.3–2.6)
55–64	14.0 (12.4–15.7)	7.8 (7.4–8.3)
65–74	17.4 (15.7–19.0)	11.9 (11.4–12.4)
75–84	29.4 (27.2–31.7)	22.6 (21.8–23.3)
85+	48.3 (43.9–52.6)	48.1 (46.5–49.6)
Sex		
Women	17.9 (16.7–19.2)	10.4 (10.1–10.7)
Men	23.1 (21.7–24.4)	13.4 (13.1–13.8)
Marital Status		
Married/Defacto	18.3 (17.1–19.4)	10.6 (10.3–10.9)
Widowed	26.9 (25.0–28.8)	19.8 (19.2–20.3)
Single	14.1 (11.7–16.5)	4.9 (4.6–5.2)
Place of Birth		
Australia	21.7 (20.1–23.2)	12.5 (12.2–12.8)
America	11.9 (7.7–16.1)	8.7 (7.6–9.9)
Asia	18.1 (16.5–19.8)	8.7 (8.3–9.0)
Africa	19.9 (14.5–25.2)	9.3 (8.2–10.4)
Europe	25.2 (23.1–27.3)	19.6 (18.9–20.3)
Pacific	12.1 (9.4–14.8)	4.5 (4.0–4.9)
Residence		
Peri-Urban	21.6 (20.0–23.2)	11.1 (10.8–11.5)
Urban	20.1 (19.0–21.2)	12.2 (12.0–12.5)
Hospital Health Insurance Cover		
Full Hospital Cover	21.5 (18.5–24.5)	20.6 (19.5–21.6)
No Hospital Cover	20.5 (19.5–21.5)	11.1 (10.9–11.4)
Episode Length of Stay (LoS)		
≤ 4 days	13.8 (12.9–14.7)	6.6 (6.4–6.8)
> 4 days	38.5 (36.2–40.9)	41.0 (40.0–42.1)
ICU Admission		
No	18.2 (17.3–19.1)	10.4 (10.2–10.6)
Yes	111.0 (97.6–124.4)	144.0 (136.7–151.2)
Primary admission diagnosis (ICD Classification)		
Nervous System	14.1 (9.3–18.9)	7.2 (6.0–8.4)
Respiratory System	47.9 (42.8–53.0)	49.9 (47.8–52.0)
Circulatory System	38.5 (35.0–42.0)	46.8 (45.0–48.6)
Digestive System	11.3 (9.3–13.3)	10.3 (9.6–11.0)
Musculoskeletal + Connective System	2.9 (1.4–4.5)	2.9 (2.3–3.5)
Skin + Subcutaneous System	4.0 (1.5–6.6)	4.8 (3.7–5.8)
Endocrine, Nutritional + Metabolic System	7.7 (4.9–10.5)	14.3 (12.1–16.6)
Number of Comorbidities		
Only Primary admission diagnosis	10.9 (4.3–26.1)	0.6 (0.5–0.6)
Primary+ one comorbidity	3.1 (1.8–4.5)	1.7 (1.5–1.9)
Primary+ 2/more comorbidities	22.0 (21.0–23.0)	23.4 (23.0–23.8)

*ICD = International Statistical Classification of Diseases and Related Health Problems; ICU = intensive care unit; CI = confidence interval*

In comparison with Europeans, Pacific-born adults had lower mortality rates in hospital, irrespective of their diabetes status. Those who stayed longer than 4 days in hospital had higher mortality rate compared with those with shorter hospital stay (1–4 days) irrespective of their diabetes status and the highest mortality rates were observed among those who received intensive care during admission (111, 95% CI 97.6–124.4 and 144 95% CI 136.7–151.2, per 1000 admitted persons with and without diabetes, respectively).

### Standard mortality ratios among admitted adults with and without diabetes in SWS

**Fig. 1:**
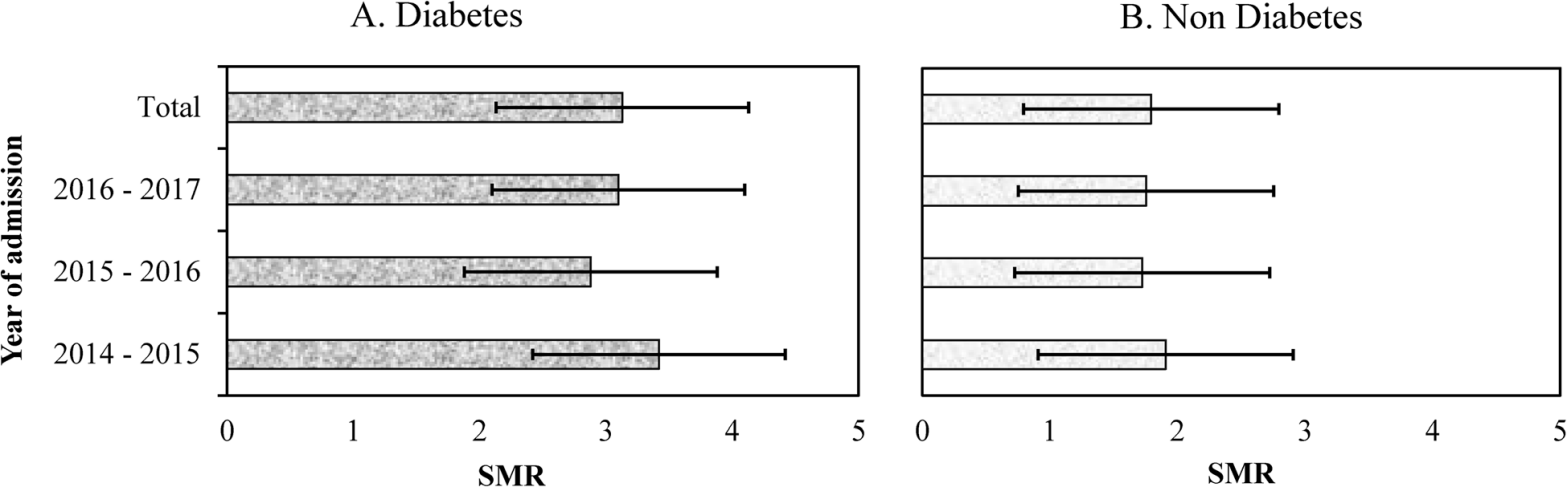
Standard mortality ratios (SMRs) in admitted adults with and without diabetes living in South Western Sydney Local Government Area of New South Wales, Australia. *Error bars are 95% confidence intervals of standard errors*

When adjusted for age and gender using the Australian population data, the SMRs (95% CIs) for people with diabetes in admission for the years 2014–15, 2015–16, and 2016–17 are presented in Fig. 1 for people with and without diabetes. The SMRs were similar over three years but were significantly greater in those with diabetes compared with the background population. Over the study period, the SMR (95% CIs) in those with diabetes was 3.13 (1.78–4.48) and 1.79 (0.77–2.82; *P* = 0.002) in those without diabetes. Over the study period, the SMR was 1.77 (0.44–2.23) in men and 1.34 (0.77–2.77, *P* = 0.230) in women with diabetes while the corresponding SMRs in men and women without diabetes were 0.93 (0.21–1.66) and 0.86 (0.14–1.57, *P* = 0.330) respectively, all of which were similar to the background population.

### Factors associated with mortality among admitted adults with and without diabetes in SWS

Table 3 presents the unadjusted and adjusted odd ratios for death during admission in both groups. The corresponding data for all patients (pooled analysis) in this study is shown in Supplementary Table 1 (S1). Among those with and without diabetes, the common factors associated with higher odds of death during admission included increasing age (55 years and over), male sex, marital status (divorced/widowed), staying longer than 4 days in admission, receiving intensive care during admission, and having a primary diagnosis that affects the respiratory or circulatory system in admission. The odds of death in admission was increased by up to 28-folds among those without diabetes who had more than one comorbidity but not in those with diabetes.

**Table 3:** Factors associated with mortality among adults with and without diabetes admitted to South Western Sydney Local Health District hospitals by diabetes status. Unadjusted (OR) and adjusted odds ratios (aOR) with their 95% confidence intervals are shown

Variables	Diabetes		Non-diabetes	
Financial year	OR (95 CI)	aOR (95 CI)	OR (95 CI)	aOR (95 CI)
July 2014–15	1.00	1.00	1.00	1.00
July 2015–16	0.87 (0.74–1.01)	0.82 (0.70–0.96)	0.93 (0.87–0.99)	0.83 (0.78–0.89)
July 2016–17	0.92 (0.79–1.07)	0.89 (0.76–1.04)	0.93 (0.88–0.99)	0.83 (0.78–0.89)
Demography				
Age groups				
18–54	1.00	1.00	1.00	1.00
55–64	2.27 (1.67–3.09)	1.92 (1.36–2.69)	3.20 (2.85–3.60)	3.72 (3.27–4.23)
65–74	2.82 (2.11–3.77)	2.40 (1.74–3.31)	4.87 (4.39–5.40)	4.94 (4.39–5.57)
75–84	4.83 (3.65–6.40)	3.69 (2.68–5.08))	9.34 (8.48–10.30)	7.23 (6.44–8.12)
85+	8.09 (6.06–10.79)	6.47 (4.67–8.97)	20.44 (18.56–22.52)	12.08 (10.76–13.56)
Sex				
Women	1.00	1.00	1.00	1.00
Men	1.29 (1.14–1.47)	1.41 (1.23–1.62)	1.29 (1.23–1.36)	1.25 (1.19–1.33)
Marital status				
Married	1.00	1.00	1.00	1.00
Widowed	1.49 (1.30–1.70)	1.21 (1.05–1.41)	1.89 (1.79–1.99)	1.08 (1.02–1.15)
Single	0.77 (0.60–0.99)	1.15 (0.88–1.51)	0.46 (0.42–0.51)	0.95 (0.86–1.06)
Place of birth				
Australia	1.00	1.00	1.00	1.00
America	0.54 (0.33–0.90)	0.51 (0.31–0.85)	0.69 (0.57–0.84)	0.80 (0.66–0.97)
Asia	0.83 (0.71–0.98)	0.87 (0.74–1.03)	0.69 (0.64–0.74)	0.96 (0.89–1.03)
Africa	0.92 (0.62–1.36)	0.93 (0.62–1.39)	0.74 (0.62–0.88)	1.06 (0.90–1.27)
Europe	1.17 (1.00–1.37)	0.91 (0.77–1.07)	1.58 (1.48–1.68)	0.96 (0.90–1.02)
Pacific	0.55 (0.40–0.77)	0.68 (0.49–0.97)	0.35 (0.31–0.41)	0.92 (0.79–1.07)
Residence				
Peri-urban	1.00	1.00	1.00	1.00
Urban	0.93 (0.81–1.06)	–	1.10 (1.04–1.16)	–
Hospital health insurance cover				
Full hospital cover	1.00	1.00	1.00	1.00
No hospital cover	0.95 (0.77–1.17)	–	0.54 (0.50–0.58)	0.86 (0.79–0.93)
Episode length of stay (LOS)				
≤ 4 days	1.00	1.00	1.00	1.00
> 4 days	2.87 (2.53–3.25)	1.77 (1.54–2.03)	6.45 (6.13–6.79)	1.61 (1.52–1.70)
ICU admission (No, OR = 1)				
Yes	6.73 (5.56–8.15)	4.97 (4.00–6.16)	15.98 (14.73–17.32)	5.40 (4.93–5.91)
Primary admission diagnosis (ICD classification)				
Nervous system (No, OR = 1)	0.67 (0.42–1.09)	–	0.60 (0.48–0.76)	–
Respiratory system (No, OR = 1)	2.69 (2.27–3.18)	2.02 (1.69–2.43)	5.15 (4.82–5.51)	2.15 (2.00–2.31)
Circulatory system (No, OR = 1)	2.18 (1.88–2.53)	1.54 (1.30–1.82)	4.95 (4.65–5.27)	1.82 (1.69–1.95)
Digestive system (No, OR = 1)	0.51 (0.40–0.67)	0.68 (0.52–0.89)	0.86 (0.77–0.95)	0.85 (0.76–0.95)
Musculoskeletal + connective system (No, OR = 1)	0.13 (0.06–0.28)	0.17 (0.08–0.37)	0.24 (0.18–0.32)	0.22 (0.16–0.30)
Skin + subcutaneous system (No, OR = 1)	0.19 (0.08–0.46)	0.28 (0.12–0.68)	0.40 (0.29–0.54)	0.41 (0.30–0.55)
Endocrine, nutritional + metabolic system (No, OR = 1)	0.36 (0.21–0.60)	0.43 (0.25–0.73)	1.22 (0.98–1.52)	–
Number of comorbidities				
Only primary admission diagnosis	1.00	1.00	1.00	1.00
Primary+ one comorbidity	0.29 (0.04–2.25)	0.08 (0.01–0.64)	3.06 (2.35–4.00)	4.23 (3.21–5.57)
Primary+ 2/more comorbidities	2.04 (0.28, 14.7)	0.32 (0.04–2.45)	43.0 (35.6, 52.0)	28.68 (23.49–35.02)

*Empty cells in the adjusted odd ratios were variables not included in the adjusted model for lack of significance. Confidence intervals (CIs) that exclude 1.00 are significant (p < 0.05). ICD = International Statistical Classification of Diseases and Related Health Problems; ICU = intensive care unit*

Although the mortality rate was greater in the diabetes group compared to the non-diabetes group, this was not seen in the 85+ age group where the adjusted odds ratio was higher in the non-diabetes group (adjusted odds ratio, aOR 12.08, 95% CI 10.76–13.56) than the diabetes group (aOR 6.47, 95% CI 4.67–8.97). In both groups, Europeans had higher odds of death in admission but this was nullified after adjusting for the potential covariates (Table 3). Also, staying longer than 4 days in hospital and having a primary diagnosis of respiratory system disease were associated with higher odds of death in the non-diabetes group than in the diabetes group.

## Discussion

The current understanding of the international burden of diabetes-related complications is poor and there is paucity of data on a population level, for hospital mortality among Australian adults with diabetes. Using the national representative sample of more than 554,000 adult admissions of people with and without diabetes from 2014 to 15 to 2016–17, our study provided the needed regional data on hospital outcomes of Australians living in the high-risk region of South Western Sydney [1, 7, 8]. The study showed that over the three years, and across all ages, people with diabetes continue to have higher mortality rates compared with their counterparts without diabetes. Diabetes inpatient SMR was 50% higher than non -diabetes SMR,and about three times higher than the general Australian population. Furthermore, the SMR was higher in men than women, and this did not improve over time. Multivariable analyses revealed similarities in the drivers of mortality in both groups, with higher odds of death on admission in the diabetes than the non-diabetes group mostly in those aged 55 years and over, who were previously married, or stayed in hospital for more than 4 days and people who received intensive care during admission. Irrespective of the diabetes status, a primary diagnosis of respiratory and circulatory system disease should be considered a red flag in hospital, since it further increases the odds of death during admission.

The study showed for the first time that the standardized mortality among admitted people with diabetes in Australian public hospitals was significantly higher than the background population and remained so after three years. This finding was consistent with previous studies in non-admitted people [4]. Harding et al’s study [4] used data from Australians who were registered on the National Diabetes Services Scheme between 1997 and 2010 but data was analysed based on diabetes type. They found that people with type 1 and type 2 diabetes experienced 3 and 1.2 times increased risk of all-cause mortality, respectively, over thirteen years [4]. In another retrospective study of patients aged 25–64 years admitted to hospitals in Perth, Australia, between 1985 and 1993 with AMI diagnosed, the authors reported that mortality was 28.1 and 12.0% for people with and without diabetes, respectively (*P* < 0.001) [10]. However, using database of all general internal medical discharge episodes at a Melbourne hospital between July 2012 to June 2013, authors found similar number of days of admission and mortality rates between those with and without diabetes [5]. Without discriminating between diabetes type, Lind et al [29] used a large diabetes population in Ontario, Canada to show that the diabetes mortality rate ratios decreased from 1.90 to 1.51 between 1996 and 2009 and decreased from 2.14 to 1.65 in a diabetes population from the U.K. Data presented from the National Survey of U.S. adult population with diabetes found that between 1997 and 2006, there was a decline of 23 and 40% in all-cause and CVD death rates respectively, which were similar between gender [30]. Allemann et al [31] found that SMRs for people with type 1 and type 2 diabetes decreased significantly from 4.5 in 1974 to 3.5 in 2005 in Switzerland. The higher SMRs reported in their study [31] compared to the present study may be related to the longer follow up periods and the fact that the previous studies were conducted when mortality from diabetes was much higher compared with the general population. In addition, the small differences with data from other studies discussed reflect differences in the populations.

This study found that men had higher mortality rate and were more likely to die in hospital compared with women, independent of the diabetes status. Mortality rates were 1.3 times as high for men as women (23.1 and 19.9 per 1000 admitted people in SWS, respectively) and the likelihood of dying in hospital was still almost twice as high for men than women with diabetes. Consistent with our finding was the 1.7 times higher mortality rates in men than women reported in the recent Australian National survey data [1]. However, Taylor et al. found a higher relative risk of death in females with diabetes compared with their male counterparts in the UK (RR 2.47, 95%CI 2.23–2.72 versus RR 1.93, 95%CI 1.79–2.07) [32], whereas among people with diabetes admitted electively to hospital, a higher additional risk of death was also found in females than males [11]. In a cohort of US and Japanese participants with and without diabetes, Liu et al. found a higher relative risk of mortality among US women with diabetes (HR 1.59 *p* = 0.01) but no difference was observed among male and females in Japan [33]. In an Australian study using NDSS database, men showed higher excess risk of mortality than women [4]. The mixed results demonstrated by the different studies, warrants further investigation into the association between sex and diabetes related mortality.

Europeans-born adults had lower hospitalisation but higher hospital mortality rates compared with Australian-born adults and both had higher mortality rates than Pacific people. A previous report from the Australian Institute of Health and Welfare found similar higher mortality rates among Europeans than their Australian-born counterparts [34]. Although, an increased prevalence of diabetes and associated complications would be expected to lead to more frequent hospitalisations, it may not always correspond with high mortality rates [34]. Low hospitalisation rates may also reflect poor management of diabetes complications rather than less complications [35]. The fact that ethnicity remained a significant factor even after adjusting for all the potential covariates in the multivariate analysis, suggests that ethnicity plays an important role in diabetes related complications among inpatients.

Age and marital status were also associated with in-hospital mortality in people with and without diabetes even after adjustment for all potential covariates. Older age is a known risk factor for mortality [15]. The higher odds of mortality among those who were widowed was consistent with previous studies [36, 37] which found about 2.2 higher risk of all-cause mortality among those who were widowed compared with married people in Iran [36]. This can be attributed to the findings from a meta-analysis that marriage or support from the spouse is associated with a reduction of up to 15% in the risk of all-cause mortality [37]. Thus suggesting that marriage may have a health protective effect in reducing stress and anxieties and promoting positive healthy behaviours, while not being married may adversely affect the health of individuals [38]. It has also been suggested that the emotional shock of losing a spouse and a lack of social support contributed to higher mortality rate in those who were widowed [39].

The present study found that participants who received intensive care during admission had the highest odds of in hospital mortality. This was similar to the findings of a systematic review and meta-analyses, which determined that diabetes is associated with an increased mortality risk in cardiac surgery patients admitted to ICU [40] and this may be related to the high blood glucose levels in ICU [39]. Although at the time of this study, HbA1_c_ has been recommended for use as a diagnostic test for diabetes in Australia [41], it was still not commonly performed during admission across SWSLHD hospitals particularly among older participants [42].

Similar to previous studies, [12–14, 32, 33] we found significant associations between comorbidities affecting the respiratory and circulatory systems and increased odds of death in people with and without diabetes. Over 90% of those with diabetes had 2 or more co-morbidities, compared with less than 50% without diabetes. Past studies [12–14] have demonstrated significant associations between mortality and presence of cerebrovascular, renal and vascular diseases, and these were replicated in our study. In addition, we found significantly higher odds of death among non-diabetes participants who had either one or two/more comorbidities documented but not in diabetes group even after correcting for the potential cofounders. Other studies have also documented that comorbid diabetes mellitus adds significantly to hospitalisation duration and costs in medical inpatients [5]. These findings the suggesting the overall poorer health outcomes in residents of South Western Sydney compared to NSW as a whole [8].

The main strength of this study is that it is population based with a large sample size. Also, the use of place of residence rather than admitting hospital to define the population removes the bias associated with secondary/tertiary care centres, as well as including people admitted to hospitals outside the district. The main limitations of the study are due to the nature of the data being used (secondary data analysis). As with administrative databases [43], the diabetes prevalence relies on accurate discharge summaries and it is very likely that diabetes is under-reported due to coding errors. Although, a previous study [44] found good agreement between self-reported diabetes and the coded diabetes, a much higher inpatient prevalence of diabetes was found in an audit of inpatients in Melbourne [45]. Due to the higher length of stay for diabetes, the point prevalence will always be higher in hospital, but not as much when datasets like that used in this study look at all admissions. Information about type of diabetes was not available, country of birth was used instead of ethnicity, and indigeneity are not routinely available. Also, the use of de-identified data did not allow for the identification of multiple admissions, and data were not available for one of the seven LGAs in the District. No body mass index or glucose data was used in this study as these were not captured in the database at the time, and socioeconomic status data were also not available. Furthermore, data measures of diabetes severity which affect mortality risk, including duration of diabetes and glycaemic control were not available, and thus could not be adjusted for [46].

## Conclusions

This population-based in-patient study provided an understanding of the burden of diabetes in the SWS region and showed that in-patients with diabetes continue to have substantially higher mortality rates than those without diabetes over three years, with up to 3 fold increase in mortality rate compared with their age matched counterparts in the general Australian population. Although there were commonalities in the factors associated with mortality in people with and without diabetes, significantly more people with diabetes had two or more co-morbidities, suggesting that hospital mortality may be driven by those with pre-existing health/comorbidities. Considering the high economic and health burden of hospital admissions, urgent measures in primary care to prevent admissions among people with multiple co-morbidities needs to be undertaken, including optimal management of diabetes to reduce complications and risk of hospital admissions. Further research into the reason for greater mortality among patients admitted in hospital in this region are needed.

## Supplementary Information

**Additional file 1: Supplementary Material S1**. Factors associated with mortality among all adults admitted to South Western Sydney Local Health District hospitals. Unadjusted (OR) and adjusted odds ratios (AOR) with their 95% confidence intervals are shown.

## Data Availability

All data generated or analysed for the purpose of this study are included in this published article and its supplementary information files. Data is also available on request from the Centre for Health Record Linkage, which is managed by NSW Ministry of Health. Available at: https://www.cherel.org.au/data-dictionaries#section1

## Abbreviations

SWS: South Western Sydney
SWSLHD: South Western Sydney Local Health District
LGA: Local Government Area
ABS: Australian Bereau of Statistics
NSW APDC: New South Wales Admitted Patient Data Collection
ICU: Intensive Care Unit
HbA1c: Glycated haemoglobin A1c
CVD: Cardiovascular disease
LoS: Length of stay
OR: Odds ratio
aOR: Adjusted odds ratio
ICD-10: International classification of diseases, tenth version
SMR: Standard mortality ratio
CI: Confidence interval
