# Supplementary material for: Analysis of in-hospital mortality among people with and without diabetes in South Western Sydney public hospitals (2014–2017)
Source: BMC Public Health. 2021 Nov 3;21:1991. doi: 10.1186/s12889-021-12120-w (PMC8567571; doi:10.1186/s12889-021-12120-w)
Supplement: Supplementary file 1 — Additional file 1: Supplementary Material S1. Factors associated with mortality among all adults admitted to South Western Sydney Local Health District hospitals. Unadjusted (OR) and adjusted odds ratios (AOR) with their 95% confidence intervals are shown. [file 12889_2021_12120_MOESM1_ESM.docx]

**Supplementary Material**

S1. Factors associated with mortality among all adults admitted to South Western Sydney Local Health District hospitals. Unadjusted (OR) and adjusted odds ratios (AOR) with their 95% confidence intervals are shown.

| Death | OR (95 CI) | aOR (95 CI) |
| --- | --- | --- |
| Financial Year |  |  |
| July 2014-15 | 1.00 | 1.00 |
| July 2015-16 | 0.92 (0.87-0.98) | 0.83(0.78, 0.88) |
| July 2016-17 | 0.93 (0.88-0.99) | 0.84 (0.79-0.89) |
| Demography |  |  |
| Age Groups |  |  |
| 16-54 | 1.00 | 1.00 |
| 55-64 | 3.53 (3.15-3.95) | 3.39 (3.01-3.81) |
| 65-74 | 5.13 (4.63-5.68) | 4.44 (3.97-4.96) |
| 75-84 | 9.18 (8.33-10.11) | 6.62 (5.94-7.37) |
| 85+ | 20.14 (18.31-22.15) | 11.29 (10.13-12.59) |
| Sex |  |  |
| Women | 1.00 | 1.00 |
| Men | 1.31 (1.25-1.37) | 1.27 (1.21-1.34) |
| Marital Status |  |  |
| Married | 1.00 | 1.00 |
| Widowed | 1.84 (1.75-1.93) | 1.10 (1.04-1.16) |
| Single | 0.47 (0.43-0.52) | 0.98 (0.89-1.08) |
| Place of Birth |  |  |
| Australia | 1.00 | 1.00 |
| America | 0.68 (0.57-0.81) | 0.73 (0.61-0.88) |
| Asia | 0.72 (0.68-0.77) | 0.92 (0.86-0.98) |
| Africa | 0.77 (0.66-0.90) | 1.02 (0.86-1.20) |
| Europe | 1.55 (1.46-1.64) | 0.94 (0.89-1.00) |
| Pacific | 0.37 (0.33-0.43) | 0.86 (0.75-0.98) |
| Residence |  |  |
| Peri-Urban | 1.00 | – |
| Urban | 1.08 (1.03-1.13) | – |
| Hospital Health Insurance Cover |  |  |
| Full Hospital Cover | 1.00 | 1.00 |
| No Hospital Cover | 0.57 (0.53-0.61) | 0.88 (0.82-0.95) |
| Episode Length of Stay (LoS) |  |  |
| ≤4 days | 1.00 | 1.00 |
| >4 days | 5.90 (5.63-6.19) | 1.64 (1.56-1.73) |
| ICU Admission |  |  |
| No | 1.00 | 1.00 |
| Yes | 14.27 (13.24-15.37) | 5.27 (4.85-5.72) |
| Primary admission diagnosis (ICD Classification) |  |  |
| Nervous System (No, OR=1) | 0.62 (0.51-0.77) | 0.84 (0.68-1.04) |
| Respiratory System (No, OR =1) | 4.79 (4.50-5.09) | 2.20 (2.06-2.35) |
| Circulatory System(No, OR =1) | 4.48 (4.23-4.75) | 1.81(1.70-1.93) |
| Digestive System(No, OR =1) | 0.81 (0.74-0.89) | - |
| Musculoskeletal + Connective System (No, OR =1) | 0.22 (0.17-0.29) | 0.22 (0.17-0.29) |
| Skin + Subcutaneous System (No, OR =1) | 0.36 (0.28-0.48) | 0.40 (0.30-0.54) |
| Endocrine, Nutritional + Metabolic System (No, OR =1) | 1.00 (0.82-1.23) | – |
| Number of Comorbidities |  |  |
| Only Primary admission diagnosis | 1.00 | 1.00 |
| Primary+ one comorbidity | 3.17 (2.45-4.11) | 4.09 (3.12-5.35) |
| Primary+ 2/more comorbidities | 42.26 (35.03-50.99) | 26.15 (21.46-31.86) |

*Empty cells in the adjusted odd ratios were variables not included in the adjusted model for lack of significance.*

*Bolded CIs are significant variables (p<0.05, not including 1.00)*
